# Effects of different straw biochars on soil organic carbon, nitrogen, available phosphorus, and enzyme activity in paddy soil

**DOI:** 10.1038/s41598-020-65796-2

**Published:** 2020-06-01

**Authors:** Yulin Jing, Yuhu Zhang, Ihnsup Han, Peng Wang, Qiwen Mei, Yunjie Huang

**Affiliations:** 1 College of Resource Environment and Tourism, Capital Normal University, 100048 Beijing, China; 2 Department of Environmental Engineering, University of Seoul, 02504 Seoul, Korea; 3 Faculty of Civil Engineering and Mechanics, Jiangsu University, 212013 Zhenjiang, China

**Keywords:** Climate-change mitigation, Climate-change mitigation, Agroecology, Agroecology

## Abstract

Biochar is widely used as a soil amendment. Enzyme activity is an important factor that reflects soil metabolic activity, and is involved in biochemical processes such as organic matter decomposition and nutrient cycling in soils. However, the effects of biochar prepared for different straw materials on soil enzyme activity and soil nutrients are rarely studied. Through pot experiments, the effects of different straw (wheat, rice, maize) biochars (obtained by pyrolysis at 500 °C) on soil organic carbon, nitrogen, available phosphorus, and enzyme activity were studied in paddy soil. The results showed that the addition of biochar increased the soil organic carbon content, which gradually decreased with the extension of the rice growth period. The soil ammonium nitrogen content gradually decreased as the rice growth period continued; however, the soil nitrate nitrogen content first decreased and then increased over the rice growth period. Soil invertase, phosphatase, and urease activity first increased and then decreased, and the enzyme activity was the highest at the heading stage of rice. At this time, there were also significant correlations between enzyme activity and carbon, nitrogen, and phosphorus levels, except in the case of soil urease activity. The geometric mean of the investigated enzyme activities was the highest after amendment with rice straw biochar. These results indicate that the response of enzyme activity to biochar depends on the biochar feedstock and the rice growth stage.

## Introduction

Biochar has been suggested as a suitable material for soil remediation to improve soil structure, soil moisture retention, soil carbon sequestration and greenhouse gas emissions reductions^1–5^. However, the mechanism of soil change due to the addition of biochar are not clear. Some researchers have shown that the response of the soil environment to the addition of biochar depends on the properties of the biochar^6,7^. Feedstock and pyrolysis temperatures are key factors influencing biochar performance^7–9^. Straw, as an agricultural residue, contains abundant nutrients. Thus, straw incorporation could maintain and potentially improve the contents of soil nutrients. If straw is directly applied to the soil, microorganisms decompose it rapidly and generate gases such as CO_2_, CH_4_, and N_2_O,resulting in nutrient losses.

Biochar applications can affect various biogeochemical processes in the soil, including carbon (C), phosphorus (P), and nitrogen (N) cycling^10–12^. The addition of biochar can promote the stabilization of soil organic carbon (SOC)^13^, and the ability of soil to maintain N and P^10,14,15^. Biochar addition alters the form of N, the subsequent N stabilization and N transformation within the soil^16^. Tan *et al*. demonstrated that when one form of nitrogen was contained within biochar residue another form was released into the soil after biochar addition^12^. Biochar-amended soil can affect organic N by converting it into mineral N (ammonium and nitrate) which is directly taken up by the plant. Biochar can serve as a reservoir for soil P, and a certain proportion of P is suitable for plant absorption^17^.

Enzymes are important biocatalysts in soil biochemical reactions, such as the formation of soil organic matter, decomposition, nutrient elements transformations, and microbial growth, and metabolism^18–21^. An enzyme is a kind of protein, therefore, all factors that affect the protein will affect the enzyme activity. Biochar addition can influence the activities of different enzymes because it affects soil gas exchange, the soil specific surface area, the soil water holding capacity and other physical and chemical properties^22,23^. Previous studies have shown changes in soil enzyme activities after the addition of wheat straw biochar created at 300, 450, and 700 °C through pot experiments, and found that biochar pyrolysis at 450 °C promoted an increase in C-cycling and N-cycling soil enzyme activities. Soil total nitrogen (TN), microbial biomass carbon (SMBC) and microbial biomass nitrogen (SMBN) are the main driving factors affecting soil enzyme activity^24^. Nevertheless, it has also been reported that SMBC, SOC, and TN had significant correlations with soil enzyme activity^25^. Zhang *et al*. also found that the biochar feedstock, C/N, and soil TN were the main influential factors for soil enzyme activity^26^. However, to date, studies on these topics have concentrated on the biochar pyrolysis temperature, the biochar application rate and the soil enzyme interactions or on the effects of different biochar materials, such as wood, manure and straw^24,27–30^. There are few reports on the effects of different types of straw biochar on soil enzyme activity and C, N and P conversion.

Straw is a typical organic material generated in China. Burning waste straw produces carbon dioxide and other greenhouse gases, which have become a global concern. It has been reported that straw might be useful for biomass production to improve soil fertility and crop productivity^18^. Therefore, the specific objectives of this study were to (1) determine whether the application of biochar has a significant impact on soil nutrients or changes the activities of soil enzymes; and (2) compare whether there is a significant difference in soil nutrients or enzyme activity undersoil amendment with different types of straw biochar.

## Materials and Methods

### Soil and biochar

In April 2017, the soil was collected from a surface of 0–20 cm depth in rice fields in Changzhou, Jiangsu Province, China. Roots and plant litter were removed from the fresh soil, and the soil was passed through 2 mm sieves. The soil chemical properties are shown in Table 1.

**Table 1:** Soil chemical properties.

SOM	TN	TP	AN	NH_4_^+^-N	NO_3_^−^N	pH
g·kg^−1^	g·kg^−1^	g·kg^−1^	g·kg^−1^	mg·kg^−1^	mg·kg^−1^	
18.85	1.19	0.64	101.52	29.25	1.08	5.6

The biochar was produced by slow pyrolysis of wheat straw, rice straw, and maize straw at 500 °C at a heating rate of 15 °C min^−1^, 2 h, with limited oxygen supply. The characteristics of the different straw biochars were measured (Table 2). Images of the biochars were taken using a high-resolution scanning electron microscope (SEM) (H-7650, HITACHI). The contents of the biochar samples were analysed using Fourier transform infrared spectroscopy (FTIR) (TENSOR27, Bruker).

**Table 2:** Characteristics of different straw biochars.

Biochar Type	TN	TP	TK	Total organic C
	g kg^−1^	g kg^−1^	g kg^−1^	g kg^−1^
Wheat straw biochar	0.4577	1.851	23.567	567.41
Rice straw biochar	0.6128	1.993	27.147	537.97
Maize straw biochar	1.1050	1.833	14.46	518.11

### Experimental design and soil sample collection

**Figure 1:**
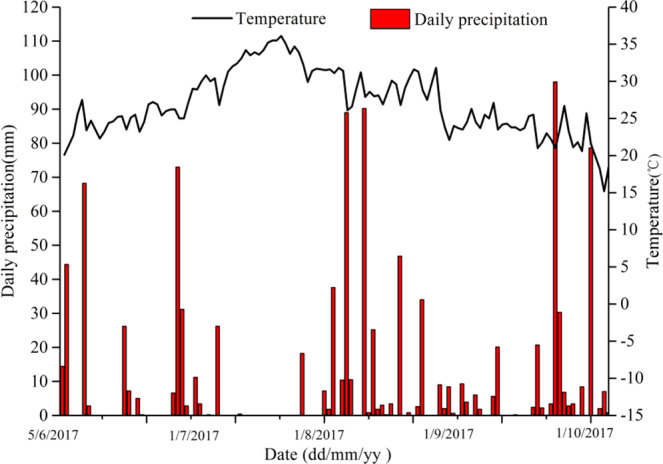
Daily precipitation and temperature during the experiment.

A natural wilderness pot experiment was conducted at Changzhou University (119.96°N, 31.69°E) in Wujin District, Changzhou City, Jiangsu Province, China, from May 19 to October 30, 2017. The climate is a north subtropical oceanic climate. The total rainfall during the rice growing season (Jun 5- Oct 30, 2018) was 973.7 mm, and the average air temperature was about 24.5 °C, ranging proximately 11.8 °C and 36.1 °C. Figure 1 shows the daily precipitation and temperature during the experiment.

The experimental design included four treatments: soil only, soil + wheat straw biochar, soil + rice straw biochar, and soil + maize straw biochar, which are referred to as CK, WSB, RSB, and MSB, respectively. The proportion of biochar in the soil was 2%. All the treatments were performed in triplicate, and the pots were arranged in a completely random block design. The pots were made of renewable polyvinyl chloride material, and each had a diameter and height of 0.3 m. The sowing and transplanting times were May 19, 2017, and June 11, 2017, respectively. For rice seed transplanting, there were 3 holes per pot and 2 seedlings per hole. The rice harvest occurred on October 30, 2017.

In all treatments, the application rate of urea was 1.89 g pot^−1^. One-third of the urea was used as a base fertilizer before transplanting, one-third was used at the tillering stage, and the remaining one third was used at the heading stage. P_2_O_5_ (1.71 g pot^−1^) and KCl (0.50 g pot^−1^) were also used as base fertilizers before transplantation. These fertilizers were evenly mixed with the soil. During rice growth, the drainage management pattern F-D-F-M was used during rice growth (flooding - drainage - rehydration - damp, during the seedling, panicle, spilling, and harvest stages, respectively). In detail, flooding was maintained in the rice pots from June 10 to July 11. The pots were drained for approximately 5 days before re-watering from July 17 to September 30, and then irrigated intermittently until harvest.

Soil samples were collected from 0 to 20 cm soil depth during the 2017 rice growth phase. The rice growth period was as follows: (A) June 11th, seedling stage; (B) July 3rd, tillering stage; (C) August 1st, elongation stage; (D) September 13th, heading and flowering stages; (F) October 30, harvest stage. The samples were sealed in plastic bags, stored on ice, and shipped to the laboratory for analysis. The samples were separated and air-dried, roots and plant litter were removed from the soil, and the soil was passed through 2 mm screens. One part of the sample was stored at room temperature for soil chemical analysis and the other was stored at 4 °C and used for enzyme activity studies.

### Soil chemical properties and enzyme activity assays

Dichromate oxidation was performed for the measurement of SOC^31^. The alkaline hydrolysis diffusion method was used for the determination of available nitrogen in the biochar. Soil NH_4_^+^-N and NO_3_^−^-N were extracted with 2 mol L^−1^ KCl at a 1:5 (m:v) ratio and determined by the indophenol blue method^32^ and the vanadium oxidation method^33^, respectively. Available P was extracted by placing 2.5 g of soil in 50 mL of 0.5 mol L^−1^ NaHCO_3_ and measured by the molybdenum blue method^34^.

The soil enzyme activities was measured based on the Guan *et al*. method^35^. The soil invertase and urease activities were measured using sucrose and urea as substrates, respectively, and incubating the soil mixture at 37 °C for 24 h. Then, the glucose and ammonia nitrogen produced were assayed using the colorimetric and indophenol blue colorimetric methods, respectively. Phosphatase activity was measured by using P-nitrobenzene as a substrate and was incubated at 37 °C for 12 h; the produced phenol was measured by the colorimetric method.

The geometric mean for the assayed enzyme activities (GMea) was calculated as GMea = (Inv × Pho × Ure)^1/3^, where Inv, Pho and Ure represent invertase, phosphatase, and urease, respectively^36,37^.

### Measurements of rice samples

Rice plants were randomly selected by choosing one seedling per hole. The plant parameters that were collected are panicle length, number of productive panicles, plant height, number of spikelets per panicle, setting rate, thousand grain weight (TGW), grain yield and straw weight. Before harvesting, the distance from the ground level to the tip of the plant was measured as the rice plant height (cm), and the panicle length (cm) was determined using a ruler. After cutting off the panicles, the straw was heated at 105 °C for approximately 1 h after which it was oven-dried at 65 °C for approximately 24 h until a constant weight was achieved. Then the straw weight was measured. The numbers of productive panicles were recorded, and the setting rate (%) was the number of productive panicles divided by the number of grains per panicle. After threshing, the grains were oven-dried at 65 °C until a constant weight was achieved; then, the TGW was recorded using an electrical balance, and the average was calculated. The grain and straw yields were determined for all plants in each experimental plot.

### Statistical analyses

Mean and standard deviation values were calculated using standard methods in Excel 2016. One-way ANOVA was used to characterize the significant effects of biochar treatment on soil SOC, N, available P, enzyme activity, and rice productivity. All statistical tests were carried out using SPSS 18.0. The Tukey test was used to assess the statistical significance of the difference between the effect of all treatments and the mean values (P < 0.05).

## Results

### SEM and FTIR of biochar

**Figure 2:**
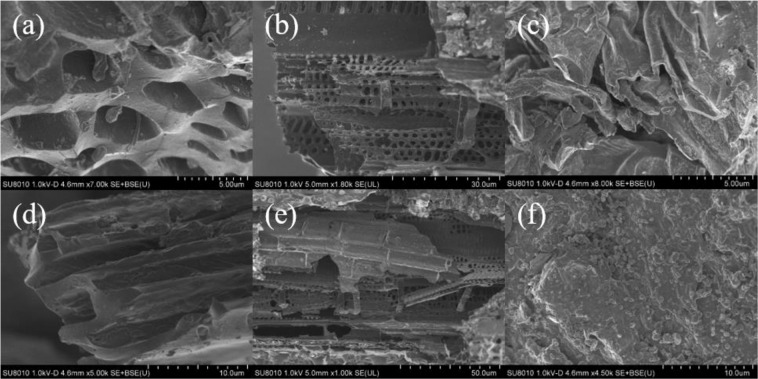
Scanning electron microscope images of wheat straw biochar (**a,d**), rice straw biochar (**b,e**) and maize straw biochar (**c,f**).

**Figure 3:**
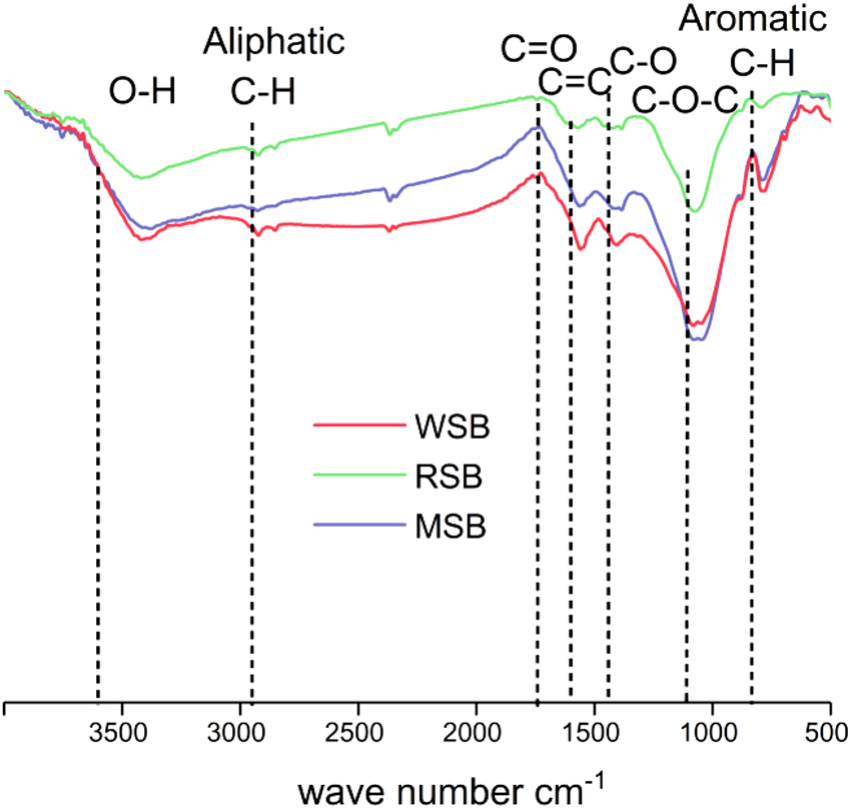
FTIR spectrum of WSB, RSB, and MSB.

The SEM image showed the change in the pores of the biochar. Figure 2 shows that the WSB sample had a smooth surface and a distinct pore structure. Some of the particulate matter adhered to the RSB sample, which had thinner rectangular structures. However, the structure of the MSB sample was slightly unclear and many particles adhered to the biological surface. The FTIR spectrum characterizes the functional groups of straw biochar. Figure 3 shows that the three samples had similar functional group structures. All had a loss of strength due to aliphatic C-H stretching and O-H stretching and had a band produced by aromatic C-H. The aromatic C = C skeleton vibration of the WSB and MSB samples had a more prominent peak than that of the RSB.

### Soil organic C, available P, and alkaline N

**Figure 4:**
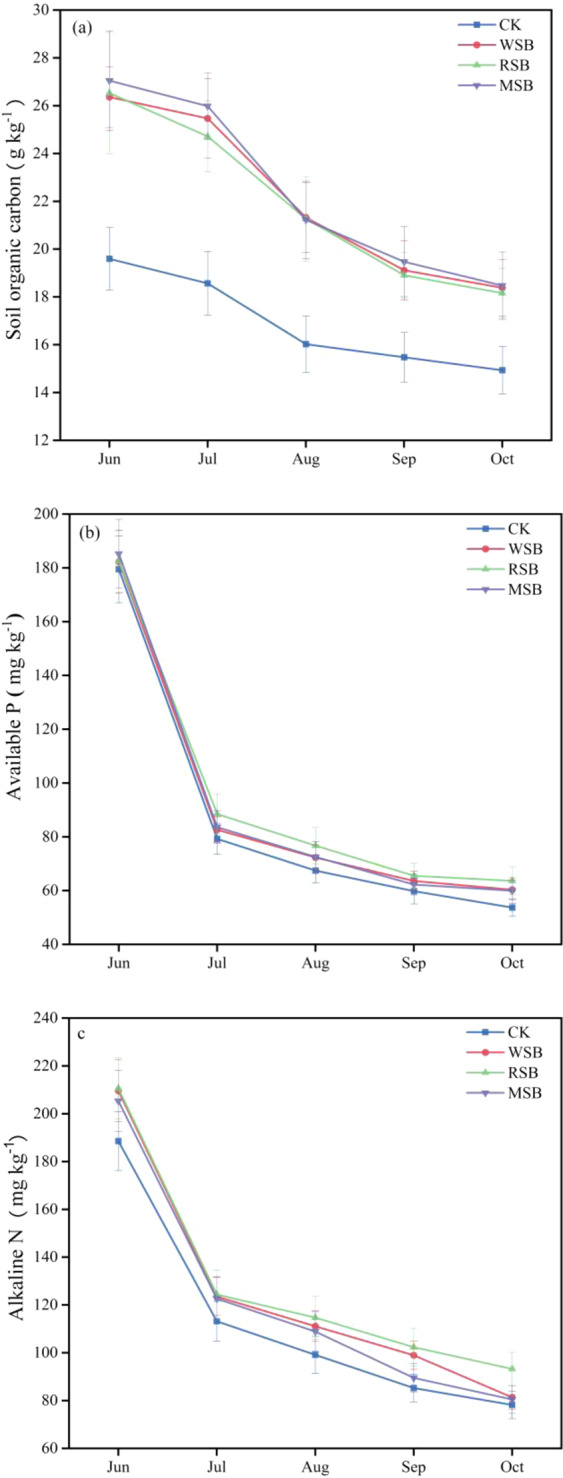
Soil organic C, available P and alkaline N content in different rice growth stages after straw biochar application in soil. (**a**) Soil organic carbon, (**b**) soil available P, and (**c**) soil alkaline N. CK: no biochar. WSB: wheat straw biochar. RSB: rice straw biochar. MSB: maize straw biochar.

During the rice growing period, the amount of SOC, available P, and alkaline N changed over time in each straw biochar treatment (Fig. 4). The three straw biochars significantly increased the SOC content compared to that in the no-biochar treatment (Fig. 4a). After 5 days of biochar application, the SOC content of each treatment was increased by 34.5~38.0% compared with that in the no-biochar treatment. There was no significant difference in SOC among the different straw biochars during the growth stages except in July. In July, the SOC content after RSB application was lower than the MSB and WSB by 1.27 and 0.75 g kg^−1^, respectively.

Figure 4b shows that the amount of soil available P changed after biochar addition to the soil during the rice growth stage. We observed that the soil available P increased with the addition of biochar, and the increase over the experimental period averaged 7.9%. In June, the available P content in MSB was 1.6% and 1.5% higher than in the WSB and RSB treatments, respectively, but, none of the treatments were significantly different (P > 0.05).

Figure 4c shows that the alkaline N levels for the RSB were above those of the other treatments at all growth stages. Averaged over the five sampling dates, the WSB, RSB, and MSB treatments increased the alkaline N content by 10.5%, 15.3% and 7.0% compared with the CK treatment, respectively. Alkaline N varied over time under the different straw biochar treatments: in September, the alkaline N content in each treatment was significantly different (P < 0.01), but no significant differences were found between the rice seedling (June) stage and the tillering (July) stage. From the seedling stage to the maturation stage, the alkaline N decreased by 58.5%, 61.1%, 55.7%, and 60.8%, under the CK, WSB, RSB, and MSB treatments, respectively.

### Soil NH_4_^+^-N and NO_3_^−^-N contents

**Figure 5:**
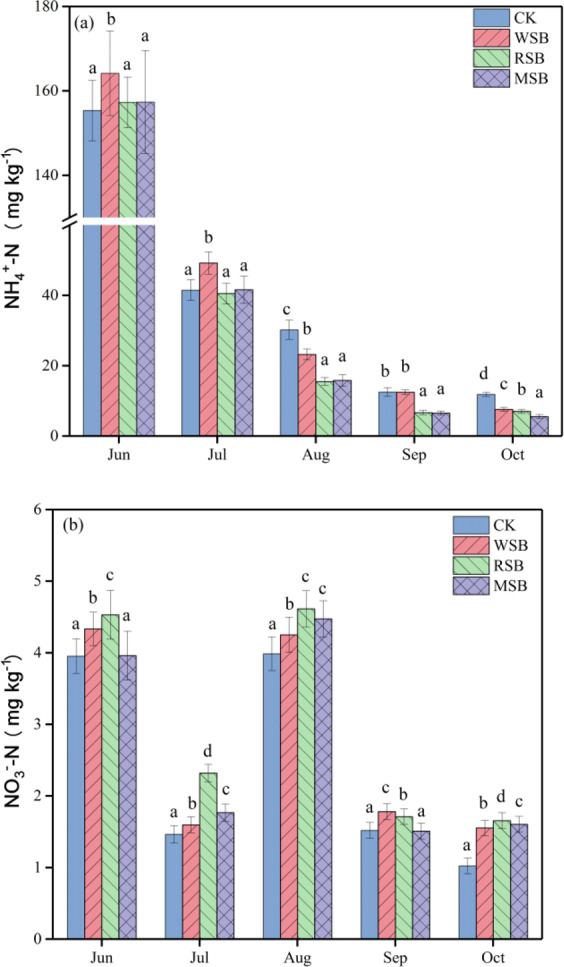
Effects of different straw biochars on soil NH_4_^+^-N and NO_3_^−^-N contents in the rice growth period. (**a**) NH_4_^+^-N, (**b**) NO_3_^−^-N. Different letters in the same time represent a significant difference by Tukey’s test at *p* = 0.05.

As shown in Fig. 5, the NH_4_^+^-N and NO_3_^−^-N contents changed differently among the different treatments. The soil NH_4_^+^-N content gradually decreased over the rice growing period for all treatments (Fig. 5a). There was a significant decrease in July compared to June, with decreases of 80.6%, 85.9%, 90.2% and 89.9% in the CK, WSB, RSB and MSB treatments, respectively. Following the addition of biochar, there was a significant increase in soil NH_4_^+^-N content compared to that in the CK treatment in June and July, whereas the CK treatment had the highest soil NH_4_^+^-N content from August to October. The application of WSB increased the NH_4_^+^-N content by 5.7% and 18.6% in June and July, respectively., compared with that in the control. However, no significant difference between the RSB and MSB treatments was evident (P > 0.05), and the soil NH_4_^+^-N content decreased based on the average of five samples from these treatments. The soil NO_3_^−^-N contents first decreased, then increased, and finally decreased during the rice growing period (Fig. 5b). The content of NO_3_^−^-N in the RSB treatment was higher than those in the CK, WSB and MSB treatments, by 12.8–61.8%, 4.5–45.3% and 3.1–31.3%, respectively, except in September. Additionally, there was a significant difference in the variations in NO_3_^−^-N content in the WSB and MSB treatments over the experimental period.

### Soil enzyme activity

**Figure 6:**
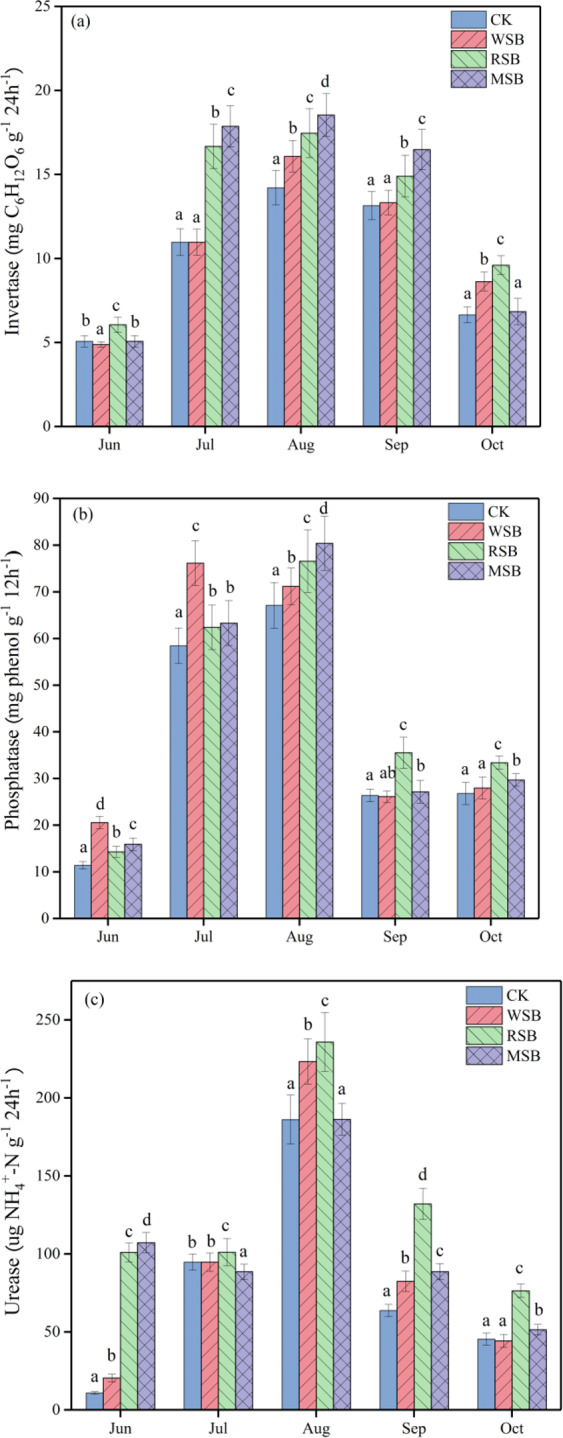
Effects of different straw biochars on the dynamic changes in soil enzyme activities during the rice the growth period. (**a**) Invertase, (**b**) phosphatase, and (**c**) urease.

The dynamic changes in invertase, phosphatase, and urease caused by different straw biochars during the experimental period are shown in Fig. 6. These enzyme activities increased first and then decreased from seedling to harvest stage and peaked in August. The activity of invertase was higher during the rice growth stage when RSB was applied, and it increased to 19.6% higher than the that in the MSB treatment in June (Fig. 6a, P = 0.000). For each sampling period, the invertase activity in the RSB treatment was higher than that in the CK treatment by 13.3–60.0%. The phosphatase activity in all treatments was higher in July and August than at other times; the WSB treatment had the highest activity in July (76.2 mg phenol g^−1^ 12 h^−1^), but the RSB and MSB treatments had the highest activity in August (76.5 mg phenol g^−1^ 12 h^−1^). In August, the urease activity of in the CK, WSB, RSB, and MSB treatments increased by 49.1–94.2%, 57.6–90.8%, 50.0–67.6% and 42.5–72.4%, respectively, compared with that at other sampling periods. The urease activity increased under the CK, WSB, and RSB treatments, but decreased under the MSB treatment from June to July. The RSB treatment had the highest urease activity during each time except June.

The geometric mean of the assayed enzyme activities (GMea) increased after applying straw biochar compared with that of the CK treatment in each rice growth stage and was highest in August (Table 3). The GMea of the RSB treatment was 7.2–62.1% and 0.4–32.8% higher than those of the WSB and MSB treatments, respectively.

**Table 3:** The geometric means for the assayed enzyme activities (GMea) of different treatments during the rice period.

Treatment	June	July	August	September	October
CK	8.57 ± 0.63a	39.30 ± 2.17a	56.19 ± 3.13a	28.06 ± 2.12a	20.30 ± 1.09a
WSB	12.71 ± 1.10b	42.93 ± 2.03b	63.47 ± 4.13b	30.62 ± 1.12b	22.02 ± 1.14b
RSB	20.61 ± 1.56c	47.18 ± 3.06d	68.04 ± 4.17d	41.19 ± 1.16d	29.04 ± 2.10c
MSB	20.53 ± 1.11c	46.45 ± 1.24c	65.24 ± 5.11c	34.09 ± 2.57c	21.86 ± 1.59b

Note: Different letters in the same period time represent significant differences by Tukey’s test at *p* = 0.05.

The results of the correlation analysis are presented in Table 4. No significant correlation was found between organic C and enzyme activity. A positive correlation was observed between the activity of invertase and the contents of NH_4_^+^-N and NO_3_^−^-N in the soil. The activities of urease, alkaline N, and available P were significantly positively correlated. By comparing the correlations between soil phosphatase activity and soil chemical properties in different periods, we found that the main factor affecting phosphatase activity was the level of soil inorganic N.

**Table 4:** Pearson correlation coefficients between soil attributes across different straw biochars in five growing seasons.

	SOC	Invertase	AP	Phosphatase	AN	NH_4_^+^-N	NO_3_^−^-N	Urease	GMea
**June**									
SOC	1	0.233	0.706^*^	0.687^*^	0.932^**^	0.464	0.493	0.677^*^	0.812^**^
Invertase		1	0.011	−0.350	0.349	−0.344	0.647^*^	0.583^*^	0.573
AP			1	0.355	0.581^*^	0.287	0.024	0.620^*^	0.658^*^
Phosphatase				1	0.705^*^	0.865^**^	0.354	−0.049	0.149
AN					1	0.568	0.696^*^	0.543	0.708^*^
NH_4_^+^-N						1	0.376	−0.269	−0.080
NO_3_^−^-N							1	0.204	0.349
Urease								1	0.976^**^
GMea									1
**July**									
SOC	1	0.567	0.610*	0.578*	0.921**	0.312	0.462	−0.136	0.819**
Invertase		1	0.644*	−0.323	0.537	−0.588*	0.697*	−0.124	0.888**
AP			1	0.023	0.734**	−0.233	0.906**	0.496	0.816**
Phosphatase				1	0.542	0.936**	−0.152	−0.056	0.080
AN					1	0.251	0.624*	0.125	0.837**
NH_4_^+^-N							−0.428	−0.112	−0.242
NO_3_^−^-N							1	0.594*	0.822**
Urease								1	0.092
GMea									1
**August**									
SOC	1	0.826^**^	0.813^**^	0.732^**^	0.905^**^	−0.842^**^	0.802^**^	0.567	0.914^**^
Invertase		1	0.728^**^	0.978^**^	0.715^**^	−0.963^**^	0.870^**^	0.175	0.869^**^
AP			1	0.671^*^	0.920^**^	−0.866^**^	0.889^**^	0.757^**^	0.953^**^
Phosphatase				1	0.625^*^	−0.940^**^	0.861^**^	0.082	0.817^**^
AN					1	−0.823^**^	0.843^**^	0.779^**^	0.943^**^
NH_4_^+^-N						1	−0.955^**^	−0.385	−0.951^**^
NO_3_^−^-N							1	0.519	0.954^**^
Urease								1	0.636^*^
GMea									1
**September**									
SOC	1	0.615*	0.675*	0.264	0.644*	−0.584*	0.437	0.559	0.566
Invertase		1	0.248	0.277	0.022	−0.911**	−0.357	0.432	0.562
AP			1	0.691*	0.887**	−0.484	0.685*	0.848**	0.786**
Phosphatase				1	0.657*	−0.640*	0.335	0.940**	0.928**
AN					1	−0.273	0.885**	0.788**	0.692*
NH_4_^+^-N						1	0.162	−0.736**	−0.835**
NO_3_^−^-N							1	0.452	0.311
Urease								1	0.986**
GMea									1
**October**									
SOC	1	0.517	0.876**	0.404	0.402	−0.926**	0.945**	0.321	0.426
Invertase		1	0.793**	0.665*	0.852**	−0.368	0.630*	0.690*	0.845**
AP			1	0.738**	0.777**	−0.818**	0.939**	0.702*	0.793**
Phosphatase				1	0.929**	−0.483	0.601*	0.984**	0.955**
AN					1	−0.397	0.593*	0.949**	0.987**
NH_4_^+^-N						1	−0.951**	−0.373	−0.418
NO_3_^−^-N							1	0.518	0.610*
Urease								1	0.970**
GMea									1

Note: SOC, AP, AN, and GMea represent soil organic C, available P, alkaline N and the geometric mean of the assayed enzymes activities, respectively.

*P < 0.05; **P < 0.01.

### Rice productivity

The impacts of the three straw biochars on rice productivity were studied (Table 5). All the rice growth parameters and rice yield components were determined to be higher in the biochar-treated soil than in the CK treatment. The MSB treatment had the highest number of spikelets per panicle (90.40), grain yield (92.49 g pot^−1^) and straw yield (97.86 g pot^−1^). The RSB treatment resulted in the highest number of productive panicles (6.90), setting rate (83.77%), tiller number (7.2) and plant height (57.95 cm). The MSB, WSB, and RSB treatments increased grain yield by 102.03%, 51.05%, and 63.04%, respectively, compared to the CK treatment. It is worth noting that among the yield parameters, the thousand-grain yield of the CK treatment was the highest, however, the differences among the treatments were not statistically significant (P > 0.05).

**Table 5:** Effect of different straw biochars on rice yield and yield components.

Treatment	Number of productive panicles	Spikelets per panicle	Setting rate (%)	Thousand-grain weight (g)	Tiller numbers	Grain yield (g pot^−1^)	Panicle length (cm)	Plant height (cm)	Straw yield (g pot^−1^)
CK	5.30 ± 0.51a	73.90 ± 3.35ab	81.53 ± 5.24a	26.53 ± 0.25a	5.40 ± 0.31a	45.78 ± 1.71a	12.75 ± 1.51a	56.63 ± 4.12a	61.98 ± 4.36a
WSB	6.20 ± 0.34b	84.80 ± 2.25b	81.88 ± 6.52a	26.33 ± 0.68a	6.10 ± 0.29b	69.15 ± 4.53b	14.53 ± 1.14b	57.42 ± 3.70a	93.42 ± 5.46b
RSB	6.90 ± 0.36b	70.70 ± 4.51a	83.77 ± 4.28b	25.37 ± 0.52a	7.20 ± 0.23c	74.64 ± 3.09c	13.67 ± 0.76ab	57.95 ± 2.66a	91.14 ± 4.49b
MSB	6.70 ± 0.43b	90.40 ± 4.91c	83.57 ± 5.67b	26.10 ± 0.12b	7.00 ± 0.52c	92.49 ± 3.61d	14.38 ± 1.23b	57.18 ± 4.92ab	97.86 ± 3.26c

## Discussion

### Impacts on soil nutrients after straw biochar application

Carbon is a significantly important nutrient that determines rice growth and productivity. The biochars contain abundant labile C because they were obtained by high-temperature pyrolysis. However, the mechanisms by which biochar affects SOC are complex^38^. Biochar can enhance soil C sequestration and reduce CO_2_ emissions. This study found that biochar had a significant impact on SOC compared with that in the no-biochar treatment. The content of SOC increased after the application of straw biochar, possibly because biochar has high organic carbon content. The MSB treatment increased the SOC content more markedly than the RSB and WSB treatments in June, possibly due to the pore structure of the MSB. We found that the outer surface profile of the MSB sample was slightly unclear, which may have affected microbial growth and subsequently influenced the mineralization of SOC^39^.

P transformation is a complicated process in soil, as it requires many steps, e.g., complexation, solubilization, and adsorption^38^. Soil available P is the index of the soil P nutrient supply level, and the content of soil available P reflects the soil storage capacity and supply capacity for P. This study showed that biochar can increase soil available P, probably because straw biochar enhances the P retention capacity of the soil. Romdhane *et al*. also found that biochar increased soluble P^40^. In addition, there were noteworthy differences among the different straw biochar amendments. Zhang *et al*. reported that biochar improved soil P availability and the extent of P input varied among types of biochar. Straw biochar contains available phosphorus, but the application amount and form of biochar types vary widely^10^. The soil available P content of MSB was higher than that of the RSB and WSB treatments in the early stage of rice growth, which may be ascribed to the higher available P content in the MSB. From June to July, the content of soil available P gradually decreased; however, the extent of the reduction was reduced after the application of biochar in comparison with that in the CK treatment. This may have been due to the biochar stimulating the transformation of organic P into available P which is released into the soil^41^.

Biochar amendments can alter the physical and chemical characteristics of soils, including the pH, oxygen content, soil moisture, and microorganisms, all of which can affect soil N transformation. Paddies with seasonal alternating wet and dry conditions may modify the form of N present in soil^42^. The transformations are mainly nitrification and denitrification in paddy soils^43,44^. In the current study, biochar application increased soil alkaline N and NO_3_^−^-N levels during the rice growing stage which may be related to the characteristics of the straw biochar. Biochar is an allogenic material that provides much nitrogen, increasing the soil N content^24^. The NH_4_^+^-N content decreased with biochar amendment, possibly because biochar has oxygen-containing functional groups and its surface is highly porous so; therefore, it can absorbs NH_4_^+^-N. Therefore, if the surface area of biochars increased, it may improve the physisorption of NH_4_^+^-N^4^. The contents of soil alkaline N, NH_4_^+^-N and NO_3_^−^-N from the seedling stage (June) to the tillering stage (July), may have varied because rice seedlings cannot generate N and can only absorb nutrients from the soil. Comparing the different straw biochars, the highest contents of NH_4_^+^-N and NO_3_^−^-N were observed in the WSB and RSB treatments, respectively. However, the MSB treatment produced the highest soil total N. This shows that soil N transformation is related not only to the biochar feedstock but also to other factors involved. For example, El-Naggar reported that the concentration of total N was increased by rice biochar application because biochar may have an indirect effect on the concentration of soil nitrogen by reinforcing the microbial communities^45^.

Significant positive correlations were found between the amounts of SOC, available P, and inorganic N (alkaline N, NH_4_^+^-N and NO_3_^−^-N). This indicates that soil C, N, and P are mutually influenced by biochar amendment.

### Effects of straw biochar on soil enzyme activity

Throughout the growth season, straw biochar application was generally found to increase the soil enzyme activity in comparison with the CK treatment. According to previous studies, the possible mechanisms by which biochar application affects soil enzyme activity are (1) biochar application changes the physicochemical characteristics of soil to influence soil enzyme activity^27^ or (2) biochar has a high specific area and porosity and absorbs substrates on its surface or blocks the reaction sites of enzymes^22,46^. In the present study, there was a positive correlation between the SOC and the soil invertase, phosphatase and urease activities, especially invertase and phosphatase activities, which had significant positive correlations at the tillering and heading stages. The peak of the soil invertase, phosphatase, and urease activities occurred during the rice heading stage. Kotroczó *et al*. observed that soil enzyme activity is stimulated by root reactions^47^. The surface of fresh biochar can provide substrates for enzymatic reactions and stimulate plant roots to exude enzymes into the soil. This is also possibly related to the soil temperature; however, further verification of this possibility is required.

In this study, the MSB treatment increased soil invertase activity more than the WSB and RSB treatments, which is consistent with changes in SOC. Therefore, we hypothesize that invertase activity increases with the higher SOC caused by biochar application. Foster *et al*. reported similar findings for C-cyclase enzymes, which are also related to the colocalization and stability of C substances and enzymes on the surface of biochar^48^. For urease activity, RSB treated soil had the highest value. Since the application of biochar accelerates N mineralization, an increase in N-cycling enzyme activity occurs^46^, as shown in the present study. Except at the seedling and tillering stages of rice, soil alkaline N and urease activity had a highly significant positive correlation. Additionally, there was a significant correlation between the soil ammonium N content and urease activity. Phosphatase participates in soil P mineralization and utilization. The highest activity of phosphatase among the three straw biochar types varied with the rice growing season and was different from patterns of invertase and urease activity. Phosphatase activity may be affected by many factors, such as soil pH and soil moisture^49^. Criquet *et al*. confirmed that phosphatases may be generated by bacteria or other microorganisms^50^. Due to the interference of high levels of phosphatase activity originating from roots, it is difficult to study the kinetics of microbial phosphatase production in soil. Therefore, the changes in soil phosphatase after biochar application are not fully understood.

GMea is a general indicator that integrates information from variables with different units and ranges of variation^36^. The GMea index is a relevant tool for estimating the effects of biochar on soil enzyme activities^37^. We observed that the added rice straw biochar increased the GMea value, indicating that rice straw biochar is beneficial for improving organic matter mineralization and soil fertility.

### Effects of straw biochar soil on rice productivity

Several studies have demonstrated that biochar applications can promote crop production^51^. The occurs because biochar amendments directly release nutrients for the rice plants. Additionally, biochar application can increase N availability to crops and decrease nutrient losses^52,53^. The current study found that the improved rice yield and yield parameters occurred because the biochar amendments had high levels of SOC, which increased N accumulation efficiency and thus improved rice production^54^. The rice grain yield results in the current study conform with those of Zhang *et al*. who also found that WSB significantly increases rice yields^51^. Kamara *et al*. also reported that rice plant height and tiller number were improved after eight weeks of RSB application^55^. Among the treatments, the RSB resulted in the highest number of productive panicles, setting rate, and tiller numbers. The grain yield of the MSB treatment was higher than that of RSB because grain yield is highly dependent on rice growth parameters, including the number of productive panicles, number of spikelets per panicle, TWG, setting rate, and tiller number. In particular, the number of spikelets per panicle is an important morpho-physiological factor that affects grain yield in rice.

## Conclusions

The current results demonstrated that WSB, RSB, and MSB exhibit different physical and chemical properties that can influence soil nutrients and enzyme activity. All straw biochars increased the SOC, available P, and alkaline N contents, which decreased over the rice growing season. The soil enzyme activities increased in soil amended with straw biochar, and there were significant differences in enzyme activity between the rice growth stages. The highest enzyme activity occurred during the rice heading stage, suggesting that the demand for nutrients is high in this stage. The enzyme activities in the maturation stage were higher than those in the seedling stage. The soil GME of the RSB amendment was the highest, suggesting that RSB can be used to enhance soil enzyme activity. The Pearson correlation analysis showed that the enzyme activity and soil nutrient levels were significantly correlated. Future research is needed on the effects of different straw biochars on soil nutrients and enzymes in long-term field studies before they are used in commercial production.
